# Abiraterone acetate versus bicalutamide in combination with gonadotropin releasing hormone antagonist therapy for high risk metastatic hormone sensitive prostate cancer

**DOI:** 10.1038/s41598-021-89609-2

**Published:** 2021-05-12

**Authors:** Takashi Ueda, Takumi Shiraishi, Saya Ito, Munehiro Ohashi, Toru Matsugasumi, Yasuhiro Yamada, Atsuko Fujihara, Fumiya Hongo, Koji Okihara, Osamu Ukimura

**Affiliations:** 1 Department of Urology, Graduate School of Medical Science, Kyoto Prefectural University of Medicine (KPUM), Kyoto, Kyoto 602-8566 Japan; 2 Department of Urology, North Medical Center, Kyoto Prefectural University of Medicine (KPUM), Yosano-Gun, Kyoto, 629-2261 Japan

**Keywords:** Prostate cancer, Prostate cancer

## Abstract

The objective of this study was to compare the efficacy of abiraterone acetate with that of bicalutamide in combination with gonadotropin-releasing hormone (GnRH) antagonist treatment for patients with high-risk metastatic hormone-sensitive prostate cancer (mHSPC). A total of 149 patients with mHSPC who underwent treatment at our hospital and affiliated hospitals between December 2013 and July 2020 were retrospectively identified. Fifty patients were administered abiraterone acetate (1000 mg/day) plus prednisolone (5 mg/day) with a GnRH antagonist (degarelix) (group A), and 99 patients were administered bicalutamide (80 mg/day) with a GnRH antagonist (group B). The prostate-specific antigen (PSA) progression-free survival (PSA-PFS) was significantly longer in group A than in group B. Abiraterone acetate therapy and Gleason score were significant independent factors of PSA-PFS. Using propensity score matching, 56 matched patients were obtained. The PSA-PFS (p < 0.001) and overall survival (OS) (p = 0.0071) of patients with high-risk mHSPC were significantly longer in group A of matched patients. Abiraterone acetate therapy and Gleason score were significant independent factors for PSA-PFS in matched patients. The PSA-PFS and OS of patients treated with abiraterone acetate in combination with a GnRH antagonist were significantly better than those treated with bicalutamide.

## Introduction

Although prostate cancer today tends to be diagnosed at an early stage due to the introduction of prostate-specific antigen (PSA) screening, some patients with prostate cancer still present metastasis at the time of diagnosis in Japan1^1,2^. The mechanism underlying prostate cancer metastasis remains unclear^3^. Androgen deprivation therapy (ADT) as systemic therapy is generally accepted as a standard of care for patients with metastatic hormone-sensitive prostate cancer (mHSPC)^4^. In Japanese clinical practice guidelines for prostate cancer, combined androgen blockade (CAB) therapy, which involves concurrent use of a gonadotropin-releasing hormone (GnRH) agonist and first-generation antiandrogen, such as bicalutamide, is recommended as the standard first-line therapy for metastatic prostate cancer. However, CAB therapy is not recommended in the National Comprehensive Cancer Network guidelines and the European Association of Urology guidelines^5^.

Recently, hormonal therapy using abiraterone acetate, a next-generation antiandrogen, was reported to improve overall survival (OS) and radiographic progression-free survival (PFS) in men with high-risk mHSPC who exhibit at least two of the following factors: Gleason score ≥ 8, at least 3 bone lesions, and the presence of visceral metastasis^6^. Although the superiority of abiraterone acetate plus prednisone with GnRH agonist over placebo with GnRH agonist for the treatment of high-risk mHSPC has been reported, there are two concerns regarding its application in clinical practice in Japan. First, as stated above, most patients with metastatic prostate cancer receive CAB therapy as first-line therapy in Japan^7^. Therefore, the effectiveness of abiraterone acetate plus prednisone with a GnRH analog should be compared with that of CAB therapy using bicalutamide. Second, the GnRH antagonist, degarelix, which does not induce a transient increase in testosterone to aggravate the symptoms, instead of GnRH agonist, is becoming a major component of ADT, especially for those who have a high metastatic burden in Japan^8^. However, there have been no reports comparing the efficacy of abiraterone acetate with that of bicalutamide in combination with GnRH antagonist treatment for high-risk mHSPC.

The aim of this study was to compare the efficacy of abiraterone acetate with that of bicalutamide in combination with GnRH antagonist treatment for high-risk mHSPC.

## Methods

### Patients and treatments

We retrospectively identified 149 patients with high-risk mHSPC at our hospital and affiliated hospitals in the KPUM Prostate Cancer Study Group between December 2013 and July 2020. All patients had two or more of the following factors: Gleason score ≥ 8, at least three bone lesions, and the presence of visceral metastasis. Fifty patients were administered abiraterone acetate (1000 mg/day) plus prednisolone (5 mg/day) with a GnRH antagonist (degarelix) according to the clinician’s preference (group A), and 99 patients were administered bicalutamide (80 mg/day) with GnRH antagonist according to the clinician’s preference (group B).

Bone and visceral metastases were assessed using bone scintigraphy and computed tomography (CT). The extent of disease (EOD) score^9^ was measured using bone scintigraphy. PSA recurrence was defined as two consecutive increases in PSA of 50% compared with nadir and ≧ 2 ng/ml on two consecutive measurement at least 1 week apart. This study was approved by the institutional review board of Kyoto Prefectural University of Medicine (ERB-C-1071-2) and of each affiliated hospital and was conducted in compliance with the Declaration of Helsinki. The institutional review board waived individual written informed consent due to the retrospective nature of this study. Opt-out information was provided to patients on the KPUM website.

### Statistical analysis

The chi-square test and Wilcoxon rank sum test were used to compare the two groups as appropriate. Kaplan–Meier analysis was used to estimate the differences in time events between the two groups using the log-rank test. Univariate analysis was performed to determine the association of abiraterone acetate therapy, age, pretreatment PSA, pretreatment alkaline phosphatase (ALP) level, Gleason score, and EOD score with PFS. Multivariable analysis was performed using Cox proportional hazard models to investigate factors associated with PFS. Abiraterone acetate therapy, Gleason score, and EOD score were included as categorical variables. Age, pretreatment PSA level, and pretreatment ALP level were included as continuous variables. Hazard ratios (HRs) and 95% confidence intervals (CIs) were calculated. Propensity score matching was used to adjust the clinical backgrounds between the two groups. Age, pretreatment PSA, pretreatment ALP level, Gleason score, EOD score, and observation period were assessed and matched at a 1:1 ratio in the two groups. Statistical analyses were performed using SAS JMP version 14 (SAS, Cary, NC, USA), and statistical significance was set at p < 0.05.

## Result

### Clinical background of the patients

Table 1 shows the clinical backgrounds of the patients. Fifty patients were administered abiraterone acetate plus prednisolone with a GnRH antagonist (degarelix) (group A). Ninety-nine patients were administered bicalutamide with a GnRH antagonist (group B). There were no significant differences between groups A and B in terms of PSA pretreatment (p = 0.554). The patients in group A were significantly younger than those in group B (p < 0.001). The pretreatment ALP level, Gleason score, and EOD score of group A were significantly higher than those of group B (p = 0.0411, 0.0439, and 0.0402, respectively), indicating that the patients in group A had more advanced and aggressive disease than those in group B. The significantly shorter observation period of group A (p < 0.001) was probably due to the late approval of abiraterone acetate in Japan. Nine patients (18%) in group A and 77 patients (53%) in group B received life-prolonging subsequent therapy after PSA progression (Supplementary Table S1). Adverse events were uncommon in both groups. Although grade 3 or 4 liver-related adverse events were reported in six patients (12%) in group A, no other serious adverse events were reported in either group.

**Table 1:** Patient characteristics.

Hormone therapy	Abiraterone acetate + GnRH antagonist (group A) (n = 50)	Bicalutamide + GnRH antagonist (group B) (n = 99)	A vs B, p-value
Median (range) age at diagnosis (years)	73.5 (53–85)	77 (57–91)	< 0.001
Median (range) pretreatment PSA level (ng/mL)	663.68 (2.72–24,201)	357.23 (4.177–32,548)	0.554
Median (range) pretreatment ALP level (IU/L)	711 (124–12,122)	519 (126–7060)	0.0411
**Pathological diagnosis**			
Gleason score 7	1	2	0.0439
Gleason score 8	8	26	
Gleason score 9	31	62	
Gleason score 10	9	9	
**EOD score**			
EOD0	3	3	0.0402
EOD1	5	23	
EOD2	13	32	
EOD3	17	23	
EOD4	11	14	
Median (range) observation period (months)	10.5 (3–23)	23 (3–88)	< 0.001

*GnRH* gonadotropin-releasing hormone, *PSA* prostate-specific antigen, *ALP* alkaline phosphatase, *EOD* extent of disease.

### Prognostic factor of PSA-PFS

As shown in Supplementary Fig. S1, the PSA-PFS of group A was significantly longer than that of group B (p < 0.001). Age, pretreatment PSA level, Gleason score, EOD score, and pretreatment ALP level has been reported as prognostic factors associated with PSA control in mHSPC treatment^10,11^. Therefore, we performed Cox logistic regression analysis to investigate factors associated with PSA-PFS in patients with high-risk mHSPC using variables of those factors in addition to antiandrogen use (abiraterone acetate or bicalutamide). Abiraterone acetate therapy (HR 7.53, 95% CI 3.48–16.30; p < 0.001) and Gleason score (HR 17.99, 95% CI 3.73–52.10; p = 0.0001) were significant independent factors for PSA-PFS in high-risk mHSPC treatment (Table 2). However, the OS of patients with high-risk mHSPC was not significantly different between the two groups (p = 0.2631) (Supplementary Fig. S2).

**Table 2:** Multivariable analysis for PSA-PFS.

	Univariate analysis			Multivariable analysis		
	HR	95% CI	p-value	HR	95% CI	p-value
Abiraterone acetate therapy	4.64	2.31–9.31	< 0.001	7.53	3.48–16.30	< 0.001
Age at diagnosis	1.89	0.60–6.07	0.28	0.42	0.12–1.55	0.19
Pretreatment PSA level	1.12	0.11–5.55	0.91	0.93	0.039–6.71	0.95
Gleason score	4.79	1.71–13.90	0.0035	17.99	3.73–52.10	0.0001
EOD score	1.68	0.78–3.64	0.18	0.282	0.57–4.27	0.40
Pretreatment ALP level	1.32	0.29–4.45	0.69	19.027	0.26–9.46	0.51

*PSA-PFS* prostate-specific antigen progression-free survival, *HR* hazards ratio, *CI* confidence interval, *EOD* extent of disease, *ALP* alkaline phosphatase.

### Difference of effectiveness of abiraterone acetate and bicalutamide for patients with high-risk mHSPC adjusted by propensity score matching

**Figure 1:**
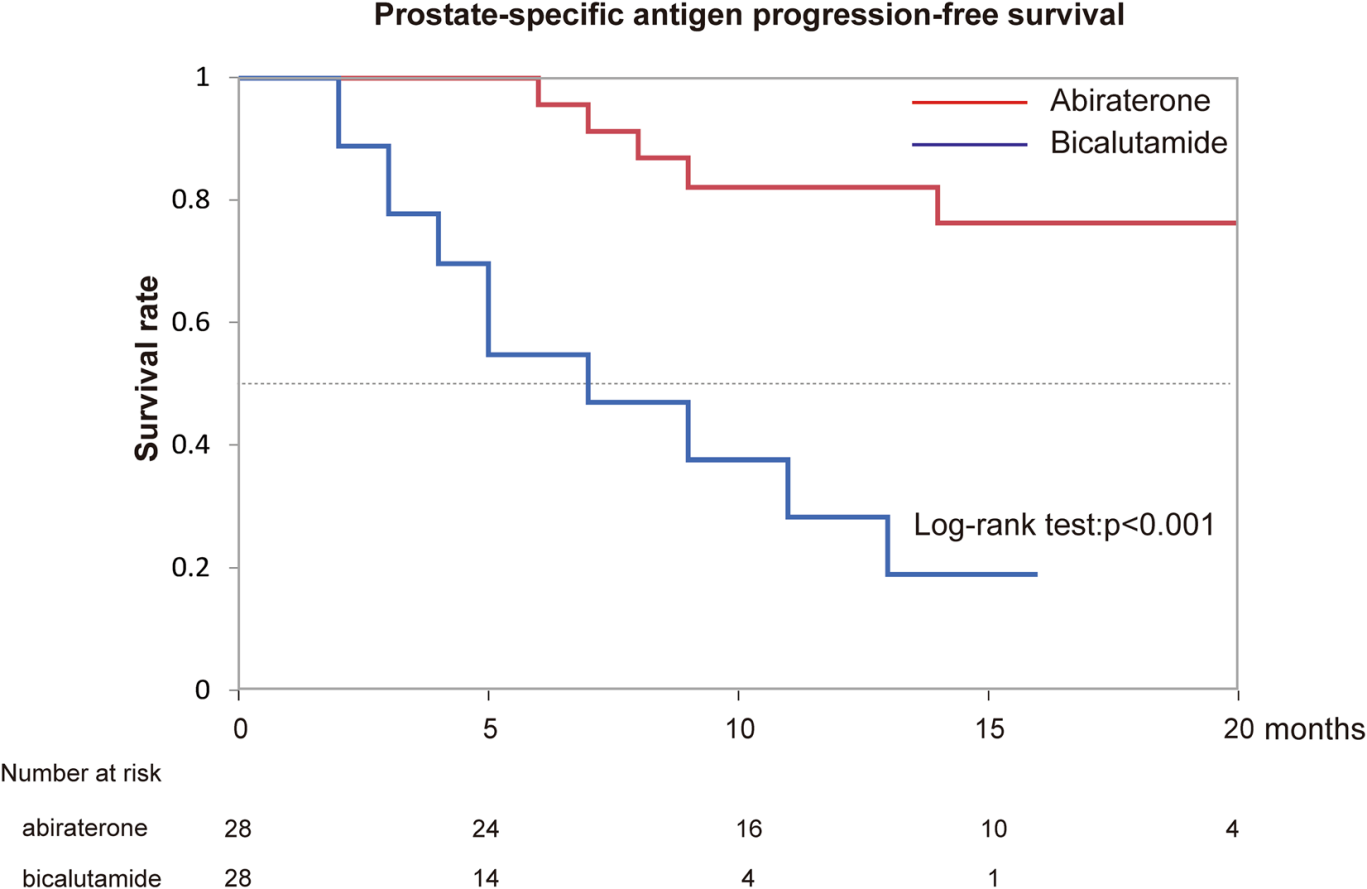
Kaplan–Meier estimates of prostate-specific antigen progression-free survival in matched patients.

**Figure 2:**
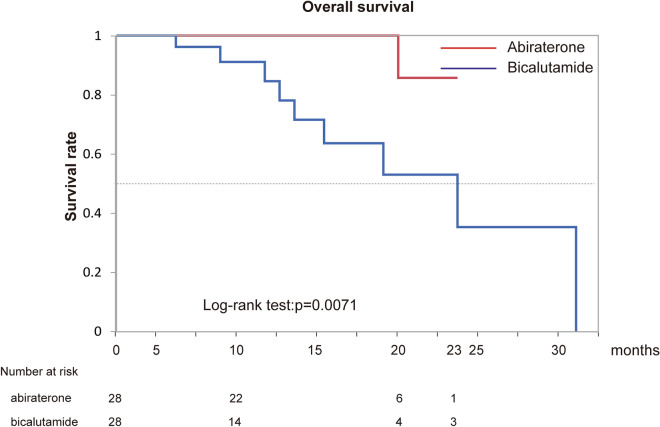
Kaplan–Meier estimates of overall survival in matched patients.

Because several factors associated with PSA-PFS and OS were significantly different between groups A and B (Table 1), we next used propensity score matching to adjust these clinical backgrounds between the two groups to examine the differences in patients with high-risk mHSPC more precisely. A total of 56 matched patients were obtained from the 149 patients. As described in Table 3, the clinical backgrounds were well adjusted between the two groups. The PSA-PFS (p < 0.001) and OS (p = 0.0071) of patients with high-risk mHSPC were significantly longer in group A of matched patients (Figs. 1, 2). Abiraterone acetate therapy remained a significant independent factor for PSA-PFS in matched patients (HR 7.09, 95% CI 2.45–20.56; p < 0.001).

**Table 3:** Characteristics of matched patients.

Hormone therapy	Abiraterone acectate + GnTH antagonist (group A) (n = 28)	Bicalutamide + GnRH antagonist (group B) (n = 28)	A vs B, p-value
Median (range) age at diagnosis (years)	74 (55–84)	76 (57–86)	0.2293
Median (range) pretreatment PSA level (ng/mL)	593.369 (10.8–10,559)	289.205 (4.177–32,548)	0.6462
Median (range) pretreatment ALP level (IU/L)	584.5 (232–3927)	731 (199–7060)	0.6128
**Pathological diagnosis**			
Gleason score 7	1	0	0.5568
Gleason score 8	4	6	
Gleason score 9	16	18	
Gleason score 10	7	4	
**EOD score**			
EOD0	1	0	0.4475
EOD1	3	6	
EOD2	10	10	
EOD3	7	8	
EOD4	7	4	
Median (range) observation period (months)	14.5 (2–23)	9.5 (3–31)	0.2093

*GnRH* gonadotropin-releasing hormone, *PSA* prostate-specific antigen, *ALP* alkaline phosphatase, *EOD* extent of disease.

## Discussion

In this study, we retrospectively compared the efficacy of abiraterone acetate with that of bicalutamide in combination with GnRH antagonist treatment for high-risk mHSPC and found that PSA-PFS and OS were significantly better in patients treated with abiraterone acetate than in those treated with bicalutamide. In our hospital and affiliated hospitals, bicalutamide appeared to be administered more frequently than abiraterone acetate to elderly patients with high-risk mHSPC, as shown in Table 1. This may be because clinicians are concerned about possible adverse events in elderly patients. However, six patients older than 80 years in group A complained of no serious adverse events. These results could lead to an increase in the number of elderly patients with high-risk mHSPC treated with abiraterone acetate.

In Western countries, CAB therapy is rarely used as the standard first-line therapy for metastatic prostate cancer, whereas in Japan, it has been widely accepted since several previous reports have suggested that Japanese patients with prostate cancer respond to CAB therapy more effectively than other races^12^. For example, it has been reported that the adjusted prostate cancer-specific mortality in Japanese patients with prostate cancer who received CAB therapy is less than half of that in American patients with prostate cancer^13^. Several reasons, such as genetic and dietary/environmental factors, have been discussed to explain the discrepancy between countries^14^. Furthermore, the difference in typical dosage of bicalutamide (80 mg/day in Japan vs 50 mg in Western countries) has also accounted for the controversial results^15^. Another report has also suggested that the dose of bicalutamide is associated with PSA response^16^. In another study, Cooperberg et al. reported that CAB therapy improved survival more significantly compared with luteinizing hormone-releasing hormone monotherapy in men with very high-risk metastatic disease, but not in those with lower-risk metastatic tumors, indicating that CAB therapy may be more effective, especially in high-risk metastatic disease than in low-risk metastatic disease^13^. One of the most important points in the current study is that we showed the superiority of abiraterone acetate use over 80 mg of bicalutamide in Japan, where these drugs are often selected for the treatment of patients with high-risk mHSPC in a real clinical setting.

Tombal et al. suggested that the GnRH antagonist degarelix may be more effective than the GnRH agonist leuprolide for PSA control^17^. Furthermore, Kashiwabara and Suda reported that GnRH antagonists were more effective than GnRH agonists for CAB treatment of bone metastatic prostate cancer with pretreatment PSA levels ≥ 50 ng/mL^8^. There are several hypotheses about why GnRH antagonist treatment results in better outcomes than GnRH agonist treatment. A previous report suggested that GnRH antagonist treatment decreases PSA levels at a faster pace than GnRH agonist^17^. This rapid effect may result in better tumor control over a longer duration. Currently, almost all patients who have been diagnosed with high-risk mHSPC in our hospital also received GnRH antagonists instead of GnRH agonists. Although this study cannot evaluate the impact of GnRH antagonists for high-risk mHSPC, comparison of PSA-PFS and OS between GnRH agonists and antagonists in CAB therapy may be studied in the future. GnRH receptor expression has been reported in various types of malignant cells, including prostate cancer cells^18,19^. Both GnRH agonists and antagonists have been reported to decrease the proliferation of prostate cancer cells^20^. The in vitro comparison of growth suppression effects between GnRH agonists and antagonists may lead to a more profound understanding of the differential effect between the two drugs.

There was a phase III study which compared the efficacy of enzalutamide, other next-generation antiandrogen, with bicalutamide in combination with ADT for mHSPC. Enzalutamide improves overall survival (OS) and progression-free survival (PFS) of men with mHSPC more significantly than bicalutamide^21^. This is the first report to compare the efficacy of abiraterone acetate with bicalutamide in combination with a GnRH antagonist for high-risk mHSPC patients in Japan. However, it is important to note that the present study has several limitations. The patient cohort was small, and there was a significant difference in patient background because of the retrospective nature of the study. Moreover, the observation period was short, especially in patients treated with abiraterone acetate. Therefore, a further prospective study with a larger cohort over a longer period is required.

In conclusion, we demonstrated that abiraterone acetate with a GnRH antagonist may have advantages over bicalutamide with a GnRH antagonist in terms of OS and PSA-PFS in patients with high-risk mHSPC. The findings in this study could provide useful information when physicians choose a treatment plan for patients with high-risk mHSPC.

## Supplementary Information

Supplementary Information.
