# Supplementary material for: Abiraterone acetate versus bicalutamide in combination with gonadotropin releasing hormone antagonist therapy for high risk metastatic hormone sensitive prostate cancer
Source: Sci Rep. 2021 May 12;11:10094. doi: 10.1038/s41598-021-89609-2 (PMC8115638; doi:10.1038/s41598-021-89609-2)
Supplement: Supplementary file 1 — Supplementary Information. [file 41598_2021_89609_MOESM1_ESM.docx]

**Abiraterone acetate versus bicalutamide in combination with gonadotropin　releasing hormone antagonist therapy for high risk metastatic hormone　sensitive prostate cancer**

Takashi Ueda^1^**^*^**, Takumi Shiraishi^1^, Saya Ito ^1^, Munehiro Ohashi^1^, Toru Matsugasumi^1^, Yasuhiro Yamada^1^, Atsuko Fujihara^1^, Fumiya Hongo^1^, Koji Okihara^2^, Osamu Ukimura^1^

^1^Department of Urology, Kyoto Prefectural University of Medicine (KPUM), Kyoto City, Kyoto 602-8566, Japan

^2^Department of Urology, North Medical Center Kyoto Prefectural University of Medicine (KPUM), Yosano-Gun, Kyoto 629-2261, Japan

**^*^Corresponding author.**

Address: Department of Urology, Graduate School of Medical Science, Kyoto Prefectural University of Medicine, Kyoto City, Kyoto 602-8566, Japan

Fax: +81 75 251 5598

Tel: +81 75 251 5595

E-mail: t-ueda@koto.kpu-m.ac.jp

**Ueda T et al., Figure S1**


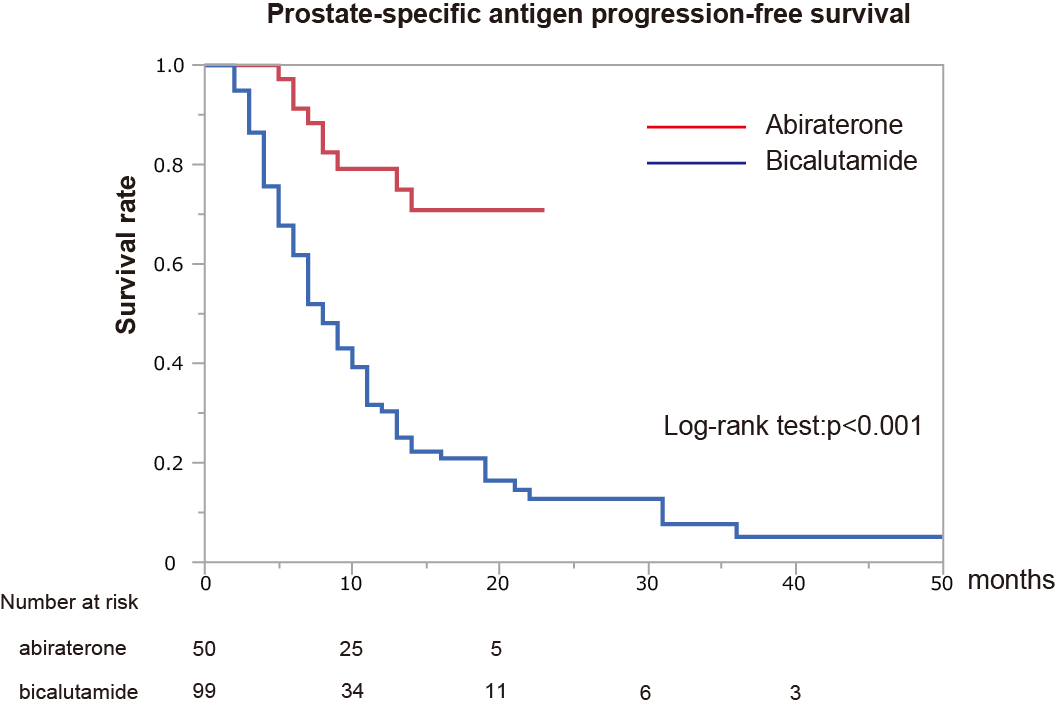


**Figure S1.** Kaplan-Meier estimates of prostate-specific antigen progression-free survival in groups A and B.

**Ueda T et al., Figure S2**
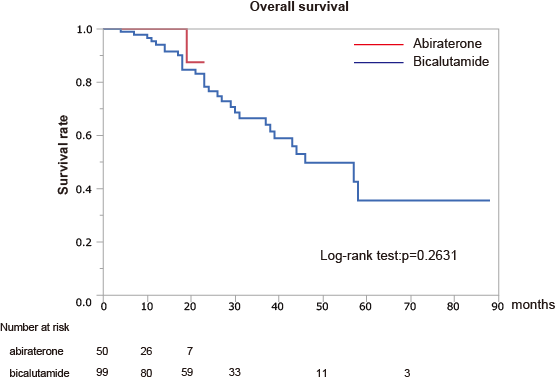


**Ueda T et al., Figure S2.** Kaplan-Meier estimates of overall survival in groups A and B.

**Ueda T et al., Table S1**

| **Agent** | **Group A (n=9, 18%)** | **Group B (n=77, 53%)** |
| --- | --- | --- |
| Abiraterone acetate+prednisone |  | 39 |
| Enzalutamide | 5 | 26 |
| Docetaxel | 4 | 12 |

**Table S1.** Sequential therapies after prostate-specific antigen progression in each group
